# The feasibility of using the EQ-5D-3L with adults with mild to moderate learning disabilities within a randomized control trial: a qualitative evaluation

**DOI:** 10.1186/s40814-018-0357-6

**Published:** 2018-10-29

**Authors:** A. M. Russell, J. L. O’Dwyer, L. D. Bryant, A. O. House, J. C. Birtwistle, S. Meer, A. Wright-Hughes, R. E. A. Walwyn, E. Graham, A. J. Farrin, C. T. Hulme

**Affiliations:** 1 Leeds Institute of Health Sciences, University of Leeds, Leeds, UK; 2 Leeds Institute of Clinical Trials Research, University of Leeds, Leeds, UK

**Keywords:** EQ-5D-3L, Learning disability, Diabetes, Economic evaluation

## Abstract

**Background:**

In trials incorporating a health economic evaluation component, reliable validated measures for health-related quality of life (HRQOL) are essential. The EQ-5D is the preferred measure for cost-effectiveness analysis in UK trials. This paper presents a qualitative evaluation of the use of the EQ-5D-3L in a feasibility randomised control trial with participants who had a mild- to  moderate learning disability and type 2 diabetes.

**Methods:**

Researchers administered the EQ-5D-3L to 82 participants at baseline and 77 at follow-up. After each interview, researchers rated the ease of administering the EQ-5D-3L and made free-text entries on the administration experience. For a subset of 16 interviews, researchers audio-recorded more detailed journal entries. Ease of administration data were analysed using descriptive statistics. Free-text responses were subject to a basic content analysis. The EQ-5D-3L-related journal entries were transcribed, coded and analysed thematically.

**Results:**

Over half of participants were perceived to experience difficulty answering some or all of the items in the EQ-5D-3L (60% at baseline; 54% at follow-up). Analysis of the free-text entries and audio journals identified four themes that question the use of the EQ-5D-3L in this population. The first theme is related to observations of participant intellectual ability and difficulties, for example, in understanding the wording of the measure. Theme 2 is related to the normalisation of adjustments for impairments, which rendered the measure less sensitive in this population. Theme 3 is related to researcher adaptation and non-standard administration. An overarching fourth theme was identified in that people with learning disabilities were viewed as ‘unreliable witnesses’ by both researchers and supporters.

**Conclusions:**

It is recommended that the EQ-5D-3L should not be used in isolation to assess health-related quality of life outcomes in trials research in adults with a learning disability. Further research is required to develop and evaluate a version of the EQ-5D appropriate for this population in trials research. It is unrealistic to expect that adjustments to the wording alone will deliver an appropriate measure: supporter or researcher involvement will almost always be required. This requirement needs to be factored into the development and administration guidelines of any new version of the EQ-5D for adults with a learning disability.

**Trial registration:**

Current Controlled Trials ISRCTN41897033 [registered 21 January 2013].

**Electronic supplementary material:**

The online version of this article (10.1186/s40814-018-0357-6) contains supplementary material, which is available to authorized users.

## Key concepts

- Adults with mild to moderate learning disability often struggled to understand the purpose of the EQ-5D-3L and what was required of them.
- Questions in the EQ-5D-3L are based on an assumption that respondents will understand key concepts underlying the questions and how these concepts, e.g. self-care, relate to quality of life.
- The lives of many people with a learning disability are organised for them so that adjustments become normalised and underlying impairments are not considered problematic.
- When present, supporters involve themselves in the process of responding and rephrasing questions or explaining concepts. Some answered for the person they were supporting. Thus a hybrid of self-completion and proxy completion tends to occur.

## Introduction

### Background

In recent years, key pieces of UK legislation have required changes in the planning and delivery of health care to address the health inequalities experienced by people with a learning disability [1, 2]. The Equality Act (2010) requires service providers to make reasonable adjustments to remove discrimination in health care and the Health and Social Care Act (2012) makes the reduction of health inequalities a legal duty. In 2016, the Accessible Information Standard required all providers of NHS and adult social care services to meet the information and communication support needs of people with a learning disability and their carers. Accessible information includes physical materials in ‘easy read’ or pictorial formats but also adjustments to direct communication with service providers to enhance understanding.

Progress in addressing inequalities for people with a learning disability in health research has been less visible. Most research systematically, and often directly, excludes participants with a cognitive impairment [3]. Research also indirectly excludes and discriminates against people with a learning disability by requiring complex consent procedures and using outcome measures that are inaccessible to, or not validated in, this population [4]. This exclusion from research further compounds the health inequalities experienced by people with a learning disability [5]. Greater inclusion of people with a learning disability in research needs more accessible procedures and materials and a range of validated accessible patient-reported outcome measures.

In the UK, decisions on health care provision must typically demonstrate value for money, which is often expressed as cost-effectiveness and based upon assessment of related quality adjusted life years (QALYS). Clinical trials of the cost-effectiveness of health interventions are required to use standardised measures of health status from which QALYs can be derived, and the EQ-5D is the preferred measure for cost-effectiveness analysis in UK trials [6, 7]. Two versions of the adult measure exist; the EQ-5D-3L and the EQ-5D-5L. Both versions have been designed for self-completion and are considered to be “cognitively undemanding, taking only a few minutes to complete” [8], page 3.

The EQ-5D-3L has five domains (mobility, self-care, usual activities, pain/discomfort, anxiety/depression) and three response options for each domain, which indicate no problems to extreme problems in relation to ‘your own health state today’ (see Additional file 1). Five domains and three levels generate 243 unique health states. The EQ-5D-5L has an increased range of response options allowing 3125 unique health states. Each health state can be converted into an index score from 1.00 (best imaginable health state) to − 0.59 (worse imaginable health state). The EQ-5D also includes a separate visual analogue scale (EQ-VAS) which records the participants’ self-rated health on a vertical 100 to 0 scale with anchors of ‘best imaginable health state’ and ‘worse imaginable health state’.

The EQ-5D-3L has been validated for use with adults with diabetes [9]. Neither the EQ-5D-3L nor EQ-5D-5L has been validated in adults with a learning disability, although the measure has been used in a number of trials where the participants are adults with a learning disability [10, 11]. The EQ-5D has been said to be superior to other preference-based measures of health status in a cognitively impaired population [12]. There are however indications that use of the measure has problems in the learning disability population. First, some studies that relied on self-completion of the measure by participants report significant levels of missing data [13]. Second, the EQ-5D has been used as a proxy measure completed by carers of people with a learning disability [14, 15]. Proxy measures of health status are always problematic to a degree, especially in relation to subjective experiences of pain and mood; however, they may be useful in conjunction with self-report measures in this population [16, 17]. Third, a version of the measure for children the EQ-5D-Y [18, 19] has also been used with adults with a learning disability although the activity examples are not appropriate for adults [20] and the EQ-5D-Y has not been validated in a population of children or adults with a learning disability. In summary, there are a number of issues with measuring the health status and health-related quality of life in adults with a learning disability and several authors have called for further research [4, 20–22].

In this paper, we report on a qualitative evaluation of using the EQ-5D-3L in a feasibility randomised controlled trial (RCT) of an intervention for people with a mild to moderate learning disability who had type 2 diabetes [23]. Despite being aware of the likely limitations of using the EQ-5D-3L, the measure was chosen as it is the preferred measure for cost-effectiveness analysis in UK and a reliable measure of general health status in adults with type 2 diabetes [9]. Our evaluation therefore goes beyond evaluating the EQ-5D-3L as a measure for people with a learning disability and provides a more detailed qualitative account of the experience of using it as a self-completion measure in a trial setting. The aim is to provide evidence to inform discussions about the development of a preference-based quality of life measure suitable for adults with a mild to moderate learning disability.

## Methods

The methods used in OK Diabetes trial and the economic evaluation are reported in detail elsewhere [24]. In brief, an individually randomised feasibility controlled trial was conducted comparing supported self-management of type 2 diabetes plus treatment as usual (TAU) versus TAU alone. Participants and their supporters were followed up for 6 months.

### Participants

The eligibility criteria were as follows: 18+ years old, with type 2 diabetes, a mild or moderate learning disability and not living in a hospital setting. Learning disability was identified by the referring clinicians or support organisations based on inclusion in a learning disability register or an assessment of functional abilities and deficits in daily activities, educational and social attainment and support needs. Assessment of mild or moderate learning disability was based on the referrer’s judgement and additionally upon the researcher’s assessment of the individual’s mental capacity to understand and consent to participation and to engage in a supported self-management of diabetes. Researchers had a conversation with participants to check that they were comfortable with their inclusion in a learning disability trial. Participants were excluded if they were on insulin (a requirement of the funding call).

### Materials

The psychometric properties of the EQ-5D require that the text of the domains and levels are used verbatim. We consulted with a founding member of the EuroQol Group to discuss whether adjustments could be made for adults with learning disability and were advised to not make changes, but to assess feasibility of administration of the measure in the RCT [25].

We supported communication by printing out each domain on a separate laminated A4 sheet using large font, with no changes made to the EQ-5D-3L text. The EQ-5D-3L was chosen over the EQ-5D-5L after consultation with the service user involvement group who felt our participants would struggle to make comparisons between the five levels.

The EQ-VAS was not included on the advice of the service user involvement group who felt completing this element of the measure would be too difficult for participants, a view supported by the findings of previous studies [11, 20]. Instead, participants were asked to rate their own health (“In general, would you say your health is?”) on a five-point scale of excellent, very good, good, fair or poor, also printed on a card using a large font.

### Procedure

The EQ-5D-3L was administered at baseline and 6 months as part of a wider research interview (see [26]). The project team including the Project Coordinator were trained in assessing mental capacity by a consultant psychiatrist. Interviewers were all experienced qualitative interviewers in health-related research who were additionally trained in assessing mental capacity and delivering the measures (including the EQ-5D-3L) one-to-one by the Project Coordinator. Each researcher had regular debriefing sessions with the Project Coordinator and completed a journal after each interview (Appendix).

For each section (domain) of the EQ-5D-3L, the large-font-laminated A4 sheet was given to the participants to read but the researcher completed the instrument. For those respondents unable to read the text on the card, the questions were read out by the researcher.

Immediately after each interview, the researcher completed a measure recording their perception of the difficulty encountered by the participant in answering the interview questions, including the EQ-5D-3L. Response options were ‘no difficulty’, ‘some difficulty’ and ‘extreme difficulty’. Researchers also recorded whether the participant’s supporter provided help with answering with any question. A free-text box was provided for further comments.

Because researchers administering the interviews at baseline had noted that participants particularly struggled to respond to the items in the EQ-5D-3L, we decided to explore these difficulties at follow-up by asking researchers to journal in more detail about completion of the EQ-5D-3L in a sample of interviews. Researchers recorded an audio journal immediately after their interview about their experiences of administering the EQ-5D-3L using a topic guide based on their experiences at baseline (see Additional file 1). Sixteen participants were sampled: eight consecutive participants with a supporter and the next eight participants without.

### Ethics

All data were stored securely at the University of Leeds accessible only to the research team, and participants were only identifiable by participant ID. Transcripts of interviews and audio journals were anonymised during the transcription process. The study received NHS ethical permission prior to commencement [12/YH/0304].

### Analysis

The data from the difficulty of administration ratings were summarised using descriptive statistics.

Free-text comments related to the ease of completion measure were analysed using a basic content analysis to gain a sense of predominant or reoccurring categories. Two authors undertook this exercise independently and met to agree categories.

The 16 journal entries were coded and subjected to thematic analysis. Two authors coded the journals, using NVivo 10 to manage the data, and agreed a coding framework; regular meetings ensured concordance in coding practice. Two other authors were involved in agreeing theme interpretation.

## Results

### Participant characteristics

At baseline, 82 participants were interviewed (51.2% female; 91.5% White Caucasian; mean age 56.4 years; SD 11.49 years).

### Ease of administering health-related quality of life measures

Table 1 illustrates the analysis of the researcher-recorded ease of administration data and supporter involvement. At both baseline and follow-up, over half of the sample had some difficulty with the health-related measures. The remainder of this paper focuses on experiences associated with the administration of the EQ-5D-3L.

**Table 1:** Ease of administration and supporter involvement

	Baseline *N* = 82	Follow-up *N* = 77
General health question		
No difficulty	58 (71%)	59 (77%)
Some difficulty	20 (24%)	14 (18%)
Extreme difficulty	4 (5%)	4 (5%)
EQ-5D-3L		
No difficulty	32 (39%)	35 (45%)
Some difficulty	39 (48%)	29 (38%)
Extreme difficulty	9 (11%)	12 (16%)
Involvement of supporter in responding		
Involvement reported	24 (29%)	16 (21%)

### Researcher free-text entries

Two main categories of researcher comments were identified from free-text entries related to the EQ-5D-3L on the data collection forms, which were made by the interviewers at baseline and follow-up (see Table 2). The first category is related to the participant experiencing difficulties understanding the language used in the EQ-5D-3L in terms of individual words, the concept behind the domains and the meaning of the response category levels. The interviewers’ free-text entries also show that they often responded to this problem by giving additional explanations to support participant understanding.

**Table 2:** Examples of EQ-5D-3L-related free-text comments recorded by researchers

EQ-5D component	Free-text comment recorded by researchers (participant number in brackets)
Mobility domain	“Although participant was able to read the cards, he initially answered he had no problems with walking. However, this had to be clarified since he’d told me that he walks using a stick…” (904) “Participant didn’t seem to understand option re - confined to bed, he said it refers to times you go to bed which varies.” (1284)
Self-care domain	“Thought self-care was using a washing machine.” (1260) “With self-care….answered ‘unable’ - this clearly wasn’t true so had to probe.” (1026) “I do wonder if he was truthful about self-care as a home help helps with this….” (1320)
Usual activities domain	“Needed to explain words….usual activities.” (1260) “Had to go through question on usual activities twice…” (1283)
Pain/discomfort domain	“Said she had no pain then seconds later she said she had constant back pain.” (1316)
Anxiety/depression domain	“Supporter changed wording of anxiety/depressed to ‘do you feel sad?’” (1043) “Didn’t understand depression & anxiety….supporter said ‘fed-up or sad.’” (1200)
Response categories	“Didn’t understand moderate & extreme… [didn’t know what they were]….” (1200)

The second category is related to the involvement of supporters, who in over 20% of cases provided explanations of the questions to the participant, provided some context for them, answered questions on the respondent’s behalf or appeared to persuade respondents to change answers to match their own perceptions of the respondent’s health state.

Supporter answered some questions because participant struggled to. Although unsure if he had been given more time, he might have been able to (1134).

### Thematic analysis of audio journals

Four themes were identified through the analysis of the researcher journal entries. The first theme is related to the observations of participant intellectual ability and difficulties in relation to (i) assumptions about health-related knowledge, (ii) ability to extrapolate from examples, (iii) understanding terminology of response levels, and (iv) concept of time. Theme 2 is related to the normalisation of adjustments for impairments. Theme 3 is related to the effects of researcher adaptation. An overarching fourth theme was identified in that people with learning disabilities were viewed as ‘unreliable witnesses’ by the researchers and supporters.

#### Observations of participant intellectual ability and difficulties

i. *Assumptions about health-related knowledge*

The wording and construction of the EQ-5D make assumptions about background knowledge and basic health ‘literacy’ that may not hold true for people with a learning disability. Even basic EQ-5D terminology may not be understood. ‘Self-care’ was a problematic phrase, and the examples used did not always help people respond in the way the researchers required:

as soon as I read out “I have no problems with self-care” he said he had some problems remembering his medication, so the example I read, “I have some problems with washing or dressing myself”, didn’t fit with the medication but it was clear to him that medication came under the heading of self-care (495).

ii. *Ability to extrapolate from examples*

There is an underlying assumption that research participants will be able to extrapolate from the examples given within the measure to their own lives. In the ‘Usual Activities’ domain of the EQ-5D-3L, examples are ‘work, study, housework, family or leisure activities’. Some of these words confused participants who required further explanation, for example, ‘leisure activities’, or help to identify personally relevant alternative examples.

when I asked him about usual activities, one of the examples I used was him going to the club .. and he told me that the club isn’t running at the moment ... so he took that quite literally, he said ‘oh well I’m not going to the club at the moment’ [so selected ‘unable to’] (1284).

iii. *Understanding use of response levels*

Assumptions of language comprehension meant the use of terminology such as ‘moderate’ and ‘extreme’ could cause confusion. Researchers responded by explaining words or altering them. However, some participants struggled to make judgements between extreme and moderate even when alternative terms were used.

She struggled most with the pain,.. we talked about the measure being about today so we tried to compare it to how she normally felt and I think she chose the moderate one in the end but I think found it difficult to say how much pain it was (91).

iv. *Concept of time*

Throughout the research, it was noted that participants had difficulty with time, for example, recalling events in a sequence or recalling the amount of activity done in a week [26]. When administering the EQ-5D-3L, researchers frequently had to remind participants that they should respond in relation to ‘how they feel right now’ and some participants found it difficult to specify this

he kept answering in quite general ways so when I was talking about pain and discomfort, he told me about how it was quite difficult for him to get up from the armchair and he uses a wheat bag to help with back pain and things like that but I kind of had to say well what about today, how are you feeling today (1284).

### Normalised adjustments

The EQ-5D domains Mobility, Self-care and Usual Activities are not always sensitive in a population with lifelong intellectual impairment and a reduced ability to manage abstract concepts [27]. For many participants, adjustments and enhanced services are the norm and limited functioning is accepted rather than considered a problem. The theme of normalised adjustments was most apparent in the questions related to the Usual Activities domain. For example, a respondent whose supporter was doing their housework because they were unable to reported they had “no problems” with housework. Participants had a specially adapted bus to pick them up from their door and drop them at a day centre, and they therefore reported ‘no problems’ with their usual activities, although without support they would have been unable to attend.

the participant had said no problems [to self-care] and the dad said well actually I help shave him (815).

### Researcher adaptation and non-standard administration

The EQ-5D-3L was administered by the researcher rather than through self-completion, and in many cases, a supporter was also present; this had several consequences. As already presented, there was evidence that researchers adapted the wording to help participants complete the measure and avoid missing data. Despite the recorded difficulties with completion of the measure, the EQ-5D-3L was incomplete for only two participants at baseline and one at follow-up. All researchers understood the nature of the feasibility study and that missing data were not a concern for the HRQoL measures. Instead, it appeared that in the one-to-one interaction researchers felt uncomfortable moving on when a participant did not appear to understand what was being asked. Researchers recorded that participants became confused or upset or became disengaged if they did not understand the question. It would appear that the researchers adapted the administration of the EQ-5D-3L to allow for this.

There was variation in the approaches taken by different researchers. Some felt it was necessary to ensure the participant understood fully and answered ‘correctly’:

On the self-care one, I read them out and she selected the bottom one [unable to wash or dress self [I am unable to wash or dress myself] and I could see this wasn’t really the case because she has a job and doesn’t really have any help with her diabetes... I read the questions again and asked if she was sure (1145).

Researchers might give further examples to participants based upon their knowledge of the person’s life from earlier in the interview. In the following quote, the researcher is giving the question personal context, by choosing something they *could and did* do from their life.

I asked what her usual activities were, we discussed that they were working at [place] and walking the dog and doing the house work and then I asked her if she found that easy or difficult or did she have some problems and we got to it that way (1145).

This approach may have led to bias by pre-selecting positive answers.

### People with a learning disability as ‘unreliable witnesses’

As evidenced from the data presented above, an overarching theme in the free-text comments and the researcher journals is that in many cases, the responses of people with a learning disability were not considered reliable. The veracity of their responses was questioned by researchers although observations were mostly related to a concern that a participant did not understand the question and so their response could not be accurate. In some cases, researchers reported that supporters over-ruled the responses of participants or felt their view was more accurate than the participants.

The supporter seemed to suggest the participant’s health was worse than the participant judged (495).

…wanted depression & anxiety split as answered 'excellent' first then anxious but not depressed. Support worker changed his mind (1134).

## Discussion

This study provides the first in-depth account of the difficulties experienced by participants with a learning disability and researchers in administration of the EQ-5D. In comparison to other studies involving adults with a learning disability, the study had high levels of participant completion rather than proxy completion. This is partially due to the questions being asked by a researcher rather than online or by post. However, despite the high self-completion and low levels of missing data, it was clear that participants experienced difficulty answering the researchers’ questions, many needed assistance to complete the measures and, in some cases, the EQ-5D-3L was effectively completed by proxy. The qualitative data provides evidence to support the view that the measure, in its current form, cannot be delivered in a standardised way to adults with a mild to moderate learning disability and that there are significant issues with the validity of the measure in this group.

A further issue is that EQ-5D norms for people with a learning disability do not exist. Overall, participants scored positively in the usual activities domain despite living what may be perceived by others as restricted lives. Independence is often linked to quality of life [28], and yet some adults with a learning disability who rely heavily on others in many aspects of their lives still have a perception of their quality of life as good. This is an argument against proxy completion and for developing an appropriate measure, method of administration and norms that enable reliable and valid self-completion of a HRQoL measure in people with a mild to moderate learning disability. Proxy completion may serve a useful function but should not be considered a replacement for self-completion. Supporters of disabled people tend to report more negatively about quality of life than the disabled person themselves [29].

Aside from the difficulties people with a learning disability may experience in terms of lower levels of literacy, there are four main factors that need to be considered when considering the use of a quality of life measure in this population. Firstly, underlying the domains and all the questions of the EQ-5D and similar measures, there are assumptions of a basic understanding of ‘quality of life’ that people with intellectual impairments may not have, or, for example, that (in)ability to wash and dress oneself is considered to have an impact on this. Second, the known difficulties that people with a learning disability have in conceptualising time or making judgements along a scale means that understanding cannot be assumed even if explained in simpler language. Third, the degree to which adjustment to impairment is normalised means the measure is not sensitive across three of the five domains of the measure (mobility, self-care and usual activities). Finally, the variation in administration and influence of researcher and supporter mean that self-completion data cannot be considered reliable.

### Limitations

The study assessed the use of the EQ-5D-3L within an interview consisting of multiple measures and questions, and the findings therefore relate to the use of the EQ-5D-3L in the context of this study. It is not possible to know if responses or experiences would be different if the EQ-5D-3L had been used as a stand-alone measure rather than part of an interview. It is possible that with a further visit or some training in how to understand the questions participants may have been more able to complete the measure. This kind of reasonable adjustment may be possible and could merit further investigation but is a very different approach to standard administration of the measures. A decision was made, after service user consultation, to use the three-level response instead of the EQ-5D-5L, and further research is required to test the feasibility of the five-level response version in this group.

## Conclusions

We propose that standard administration of the EQ-5D-3L in its current form, is not appropriate for use in trials with participants with learning disabilities. The measure relies too much on assumptions about cognitive abilities such as ability to extrapolate from offered examples to personal experience and ability to make comparisons—between health states and across time. Although supporter accounts are often used as proxies, the discrepancies between what they say and what participants say cannot be resolved in a consistent way.

The standard approach to resolving these dilemmas when talking with somebody with a learning disability is to modify the communication style—for example, changing phrases and using different examples in an attempt to chime with the participant’s experiences and understanding. However, it is not possible to do that with EQ-5D and still maintain its standing as a standardised ‘gold standard’ research measure.

To include people with a learning disability in trials with an economic evaluation component, further work is required to develop an appropriate measure of HRQoL. We anticipated that participants would struggle with understanding some of the terminology, and to conduct the study ethically we accepted that some individuals would need additional support. However, our findings strongly suggest that just simplifying the language of the measures—or for example using the EQ-5D-Y—would not overcome the most significant concerns around validity. As demonstrated by our findings, a person may understand ‘taking care of yourself’ (EQ-5D-Y) more easily than ‘self-care’ (EQ-5D-3L/5L) but would still have difficulties abstracting from examples given or with the normalisation of adjustments. Supporter or researcher involvement will almost always be required and must be reflected in administration guidelines of any new version of the EQ-5D for adults with a learning disability.

### Additional file

Additional file 1: EQ-5D-3L [30]. UK (English) © 1990 EuroQol Group EQ-5D™ is a trade mark of the EuroQol Group. (DOCX 376 kb)

## Appendix

### Topic guide for EQ-5D-3L journaling

Think about and journal issues in the following areas in each of the five questions the general health question and the introduction to the measure.

- Literacy
- Did participant read it themselves—did they also understand all of what they were reading—how much—which parts?
- Understanding of words and phrases
- Understanding of each domain
- Reporting problems
- Asking supporter or interviewer what they think
- Pointing to random statements
- Answering the first one when they have not read/heard the others
- Does it fit with what we already know from the interview so far and other visits?
- Acceptability
- Discomfort/emotional reaction to the questions
- Supporter problems
- Interference
- Protection of patient
- What works better
- What words/phrases/techniques tried at interview
- What worked in interview
- What else might work

Overall

- How long did it take
- Timing of questions—measure is about feeling *today*—how is that understood and responded to?
- Did participant get bored, change the subject or digress and was this any more so than in the rest of the interview?
