# Supplementary figures and images for: The feasibility of using the EQ-5D-3L with adults with mild to moderate learning disabilities within a randomized control trial: a qualitative evaluation
Source: Pilot Feasibility Stud. 2018 Oct 29;4:164. doi: 10.1186/s40814-018-0357-6 (PMC6205783; doi:10.1186/s40814-018-0357-6)

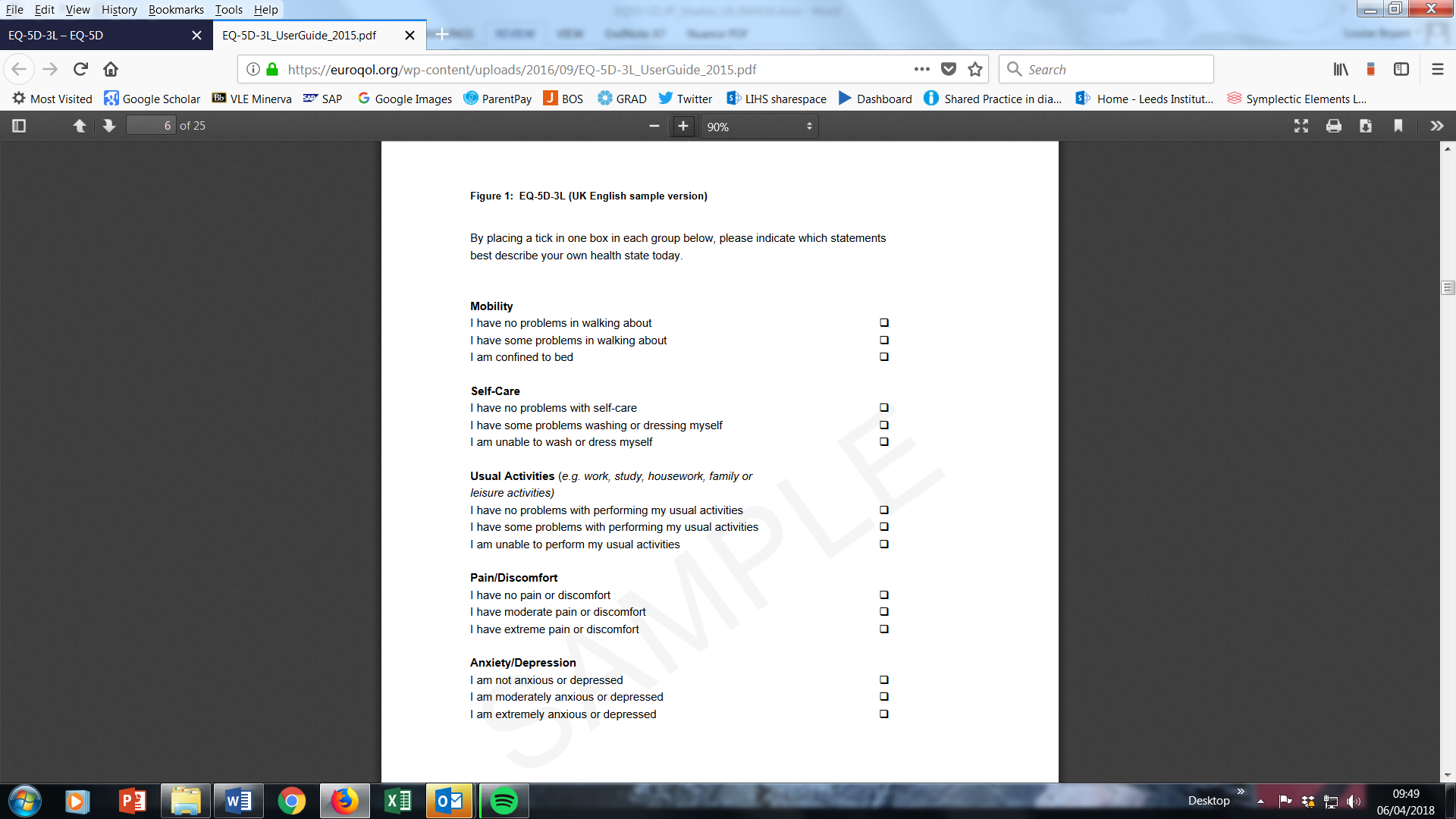

Supplement: Supplementary file 1 — EQ-5D-3L [30]. UK (English) © 1990 EuroQol Group EQ-5D™ is a trade mark of the EuroQol Group. (DOCX 376 kb) [file 40814_2018_357_MOESM1_ESM.docx]
